# Regulation of Motility of Myogenic Cells in Filling Limb Muscle Anlagen by Pitx2

**DOI:** 10.1371/journal.pone.0035822

**Published:** 2012-04-27

**Authors:** Adam L. Campbell, Hung-Ping Shih, Jun Xu, Michael K. Gross, Chrissa Kioussi

**Affiliations:** 1 Department of Pharmaceutical Sciences, College of Pharmacy, Oregon State University, Corvallis, Oregon, United States of America; 2 Department of Pediatrics, Department of Cellular and Molecular Medicine, University of California San Diego, La Jolla, California, United States of America; University of Minnesota Medical School, United States of America

## Abstract

Cells of the ventrolateral dermomyotome delaminate and migrate into the limb buds where they give rise to all muscles of the limbs. The migratory cells proliferate and form myoblasts, which withdraw from the cell cycle to become terminally differentiated myocytes. The myogenic lineage colonizes pre-patterned regions to form muscle anlagen as muscle fibers are assembled. The regulatory mechanisms that control the later steps of this myogenic program are not well understood. The homeodomain transcription factor Pitx2 is expressed specifically in the muscle lineage from the migration of precursors to adult muscle. Ablation of Pitx2 results in distortion, rather than loss, of limb muscle anlagen, suggesting that its function becomes critical during the colonization of, and/or fiber assembly in, the anlagen. Microarrays were used to identify changes in gene expression in flow-sorted migratory muscle precursors, labeled by Lbx1^EGFP/+^, which resulted from the loss of *Pitx2*. Very few genes showed changes in expression. Many small-fold, yet significant, changes were observed in genes encoding cytoskeletal and adhesion proteins which play a role in cell motility. Myogenic cells from genetically-tagged mice were cultured and subjected to live cell-tracking analysis using time-lapse imaging. Myogenic cells lacking Pitx2 were smaller, more symmetrical, and had more actin bundling. They also migrated about half of the total distance and velocity. Decreased motility may prevent myogenic cells from filling pre-patterned regions of the limb bud in a timely manner. Altered shape may prevent proper assembly of higher-order fibers within anlagen. *Pitx2* therefore appears to regulate muscle anlagen development by appropriately balancing expression of cytoskeletal and adhesion molecules.

## Introduction

During embryogenesis the paraxial mesoderm along the dorsal-ventral axis undergoes segmentation giving rise to the somites. These somites further differentiate to give rise to the dermomyotome and the sclerotome. The dermomyotome is subdivided into the epaxial and hypaxial dermomyotomes, and is the source of muscle progenitor cells that will form the deep back and lateral trunk musculature. Cells of the hypaxial dermomyotome delaminate and migrate to the regions of presumptive muscle group in the developing limbs. Formation of limb migratory muscle progenitor (MMP) cells begins when inductive cues from the lateral mesoderm and surface ectoderm synergistically induce the expression of Lbx1 within the ventrolateral Pax3 expression domain of dermomyotomes at limb levels [1]. The lateral mesoderm also provides signals that repress myogenesis in limb level dermomyotomes [2], and promote their delamination [3], [4] and migration [5] into the limb bud. Lbx1 expression in mice begins in the dermomyotome lips at E9.25 at forelimb levels, and is required for lateral migration. The dorsal and ventral muscle masses of E10.5 mouse limb buds consist of Lbx1^+^/Pax3^+^ limb muscle progenitor (MP) cells [6]. Numerous Lbx1^+^/Pax3^+^ myogenic cells persist in all limb muscle anlagen until at least E12.5. In the period between E11 and E12.5 the muscle masses enlarge, split and ultimately become the muscle anlagen, which resemble the adult muscles in shape and position with respect to bone anlagen. MP cells proliferate undergo withdrawal from the cell cycle and become terminally differentiated myocytes Pax3 and Lbx1 have generally been placed at the beginning of myogenic progression and activation of the Muscle Regulatory Factors (MRFs) in the embryonic limb because they are expressed earlier and their mutation leads to a loss of migratory precursors before MRFs are normally expressed [6], [7], [8], [9], [10]. These myocytes fuse with each other to form multinucleated myotubes and muscle fibers. The precise regulatory mechanisms that control each step of the myogenic program are not well understood to date.

MP cells must maintain adhesion throughout morphogenesis in order to develop into terminally differentiated muscle [11]. In order to migrate efficiently, the migrating cell must orientate the internal cellular machinery to a highly polarized, locally segregated, tightly regulated, and rapidly adaptable entity that can be rearranged in a coordinated manner. Migration occurs in a cyclical process, beginning with an external signal such as a growth factors, chemokines, mechanical forces, and ECM proteins. This leads to polarization and protrusion of the cell membrane with actin rich structures such as the broad lamellapodia or spike like filopodia, in the direction of movement. These protrusions are stabilized with a variety of adhesion proteins (integrins, syndecans, cadherins, and cell adhesion molecules) attaching the protrusion to the substratum. Adhesions serve as points of traction and of regulatory signaling to control adhesion dynamics and protrusion of the cell membrane [12]. The successful attachment to the substratum unmasks intracellular regions of the adhesion molecules to allow multiprotein complexes, termed the adhesome, to cross-link the adhesion molecule to the cytoskeleton [13]. There are several cross-linking proteins such as talin, vinculin, and alpha-actinin [14], [15], [16]. In the central and rear regions of the migrating cell the actin filaments organize themselves into thick bundles called stress fibers which terminate at both ends at the focal adhesions connected to the extracellular matrix ECM [17]. Disassembly of the adhesions is accompanied by inward movement of the cell edge and dispersal of the adhesion structures. This well orchestrated process maintains the appropriate cell–cell contacts between migratory muscle progenitor cells, controls the architecture of individual muscles and influences the ultimate shape, size and physiological function of the muscle organ system.

The bicoid–related homeobox gene Pitx2 is expressed in the lateral plate mesoderm and in muscle anlagen in all stages of myogenic progression [18], [19]. Pitx2 contributes to the establishment of network kernels that specify pre-myogenic progenitors for extraocular and mastication muscles [20]. Ablation of Pitx2 causes lethality in the mouse at E10.5–E14.5 with axial malformations, open body wall, heart defects, and arrest of organ development [21], [22], [23], [24]. Pitx2 is positioned downstream of both Wnt and growth factor signaling pathways in skeletal myogenesis and promotes muscle progenitor proliferation by direct regulation of the expression of a number of cyclin-dependent kinases [25]. Alternatively, Pitx2 represses T-box genes by recruiting corepressors and HDACs [26] and activates Hox genes during abdominal wall development (Eng et al., unpublished data).

The exact source, timing, and migration patterns of the muscle progenitors have recently been described using classic lineage tracing techniques in embryos. In this study, we identified genes that are regulated by Pitx2 in the Lbx1^EGFP^ myogenic cells by gene expression arrays in flow-sorted cells. Several genes involved in cell migration, adhesion and motility have been identified as Pitx2 targets, including microtubule stabilization, actin cross-linking, and tubulin related and intermediate filament associated genes. Data from these studies suggest that myogenic cells have large single protrusions with a highly directed migration by continuous remodeling of their cytoskeleton and stabilization of their adhesion to the ECM. Pitx2 can regulate myogenic cell migration by influencing their polarity and shape by restricting the microtubule growth and providing membrane and associated proteins needed for forward protrusion, fusion and muscle formation.

## Results

### Deformed and Reduced Appendicular Muscle Anlagen in Pitx2 Mutants

**Figure 1:**
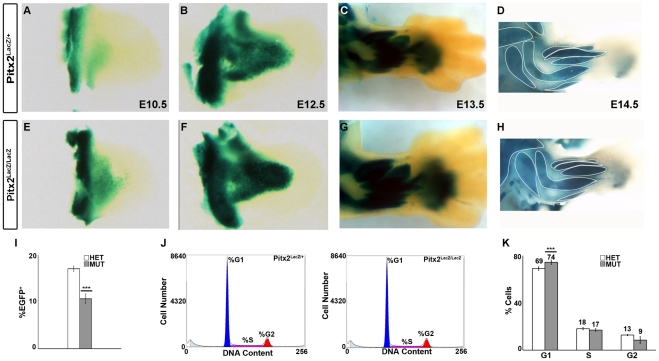
Regulation of Shape and Size of Limb Muscle Anlagen by Pitx2. (**A–H**) Whole mount X-gal staining of Pitx2^LacZ/+^ and Pitx2^LacZ/LacZ^ knock-in mice from E10.5–E14.5. Pitx2 was expressed throughout muscle anlagen but not in epidermis, mesenchyme or bone anlagen. Most distal limb muscle anlagen were mildly deformed. Muscle groups are outlined. (**I**) Percentage of EGFP^+^ cells collected from Pax3^Cre^|ROSA^EGFP^|Pitx2^LacZ/+^ (HET) and Pax3^Cre^|ROSA^EGFP^|Pitx2^LacZ/LacZ^ (MUT) embryos at E12.5 forelimb tissue dissociated into single cell suspension. The percentage of EGFP^+^ cells had a mean ± standard error of the mean (SEM) of 17±0.6% for HET (n = 8) and 11±1% for MUT (n = 7). This 26% reduction in EGFP^+^ cells in the MUT forelimb was considered to be significant using unpaired t-test with a p-value = 0.0001. (**J**) Example histograms of propidium iodide (PI) staining of HET and MUT EGFP^+^ cells isolated from forelimb tissue at E12.5. (**K**) Results of cell cycle analysis using PI staining showing distribution (mean ± SEM) of the EGFP^+^ cell population between HET (n = 5) and MUT (n = 4) during G1 (69±0.007% HET, 74±0.009% MUT); S (18±0.005% HET, 17±0.006% MUT); and G2 (13±0.003% HET, 9±0.015%MUT) phases. The increase in MUT cells during G1 phase was determined to be significant using unpaired t-test with a p-value  = 0.0102.

Analysis of E10.5–E14.5 X-gal stained Pitx2 HET embryos revealed many patches of localized blue staining in regions between the skin and bone. These patches have the pattern of muscles in the forelimbs suggesting that Pitx2 is normally expressed in muscle anlagen (Fig 1A–H). Intense X-gal staining was observed in scattered spots throughout each anlage with a more diffuse low-level stain permeating the entire anlagen. A fibrous muscle-like texture was observed in the larger stained anlagen and regions between the anlagen were not stained. The tight spatial restriction of Pitx2 expression to the muscle anlagen suggests that Pitx2 plays a role in muscle development, differentiation, and/or mature function.

The limb muscle anlagen of Pitx2^LacZ/+^ (HET) were compared with those of Pitx2^LacZ/LacZ^ (MUT) at stages E10.5–E14.5 (Fig 1), E14.5 being the latest stage possible, as MUT do not live past E14.5 due to failure of the body wall to close. At this crude level of analysis, it appeared that most, if not all, limb muscle anlagen had formed. Thus, Pitx2 was therefore not essential for the gross patterning of limb muscle anlagen. The right forelimb was the least distorted of all limbs in MUT, being only slightly pronated. Although all the appropriate muscle anlagen appeared to be present in this limb (Fig 1D,H), careful inspection revealed some differences in the shape of muscle anlagen. They appeared to be either fatter or thinner, and less finely fibered than corresponding anlagen in HET. The differences were not linked in an obvious way to the slight overall distortion of the limb in this area.

Using flow sorting we isolated EGFP^+^ cells from Pax3^Cre^|ROSA^EGFP^|Pitx2^LacZ/+^ (HET) and Pax3^Cre^|ROSA^EGFP^|Pitx2^LacZ/LacZ^ (MUT) embryos at E12.5 forelimb tissue dissociated into single cell suspensions. From forelimb tissue collected the mean (± SEM) percentage of EGFP^+^ cells was 17±0.6% in HET (n = 8) and 11±1% in MUT (n = 7) tissue mean (Fig 1I). The mean percentage of EGFP^+^ cells present in MUT tissue was reduced by 26% compared to HET and this reduction was determined significant using unpaired t-test (p = 0.0001), (Fig S1). The reduced number of cells at E12.5 may have been due to altered cell cycle in the cells isolated from MUT forelimb tissue. These EGFP^+^ cells were stained with propidium iodide (PI) and the distribution of the cells in the cell cycle showed that MUT cells had an increase in G1- phase (74±0.009%) of the cell cycle compared to those isolated from HET (69±0.007%) forelimb tissue this difference was determined significant using unpaired t-test (p = 0.0102) (Fig 1J,K), suggesting that Pitx2 regulates exit from the cell cycle. Pitx2 therefore appeared to influence the shape of limb muscle anlagen and the hypothesis was advanced that Pitx2 played a role in the proper differentiation or growth of appendicular muscle.

### Pitx2 Target Genes in the Lbx1 Myogenic Cell Lineage

**Figure 2:**
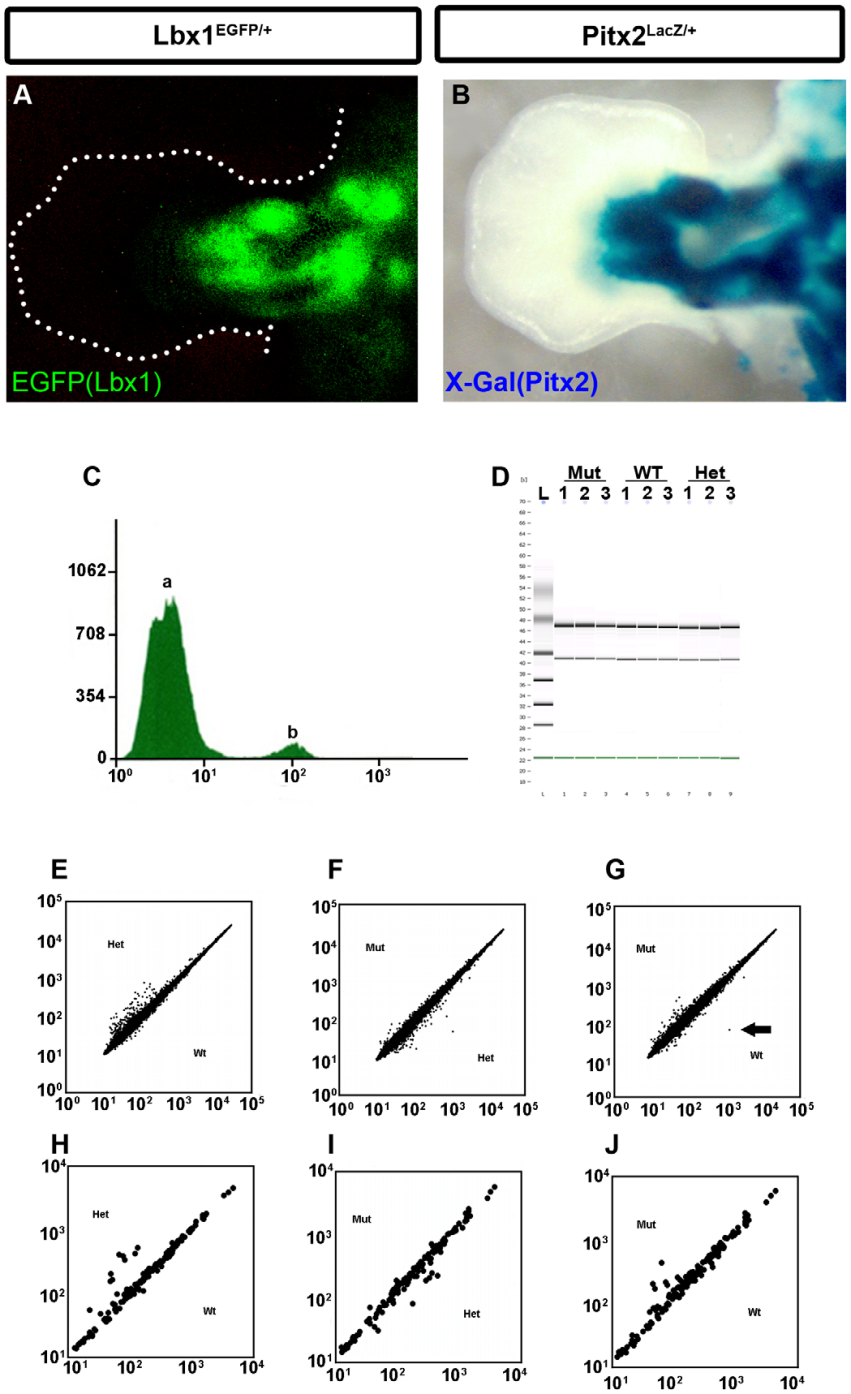
Flow-Sorting EGFP^+^ MMP Cells from Forelimbs. (**A**) EGFP indicating Lbx1 expression in Lbx1^EGFP/+^ E12.5 mouse. (**B**) Immunohistochemistry of cross-sectioned Lbx1^EGFP/+^|Pitx2^LacZ/+^ E12.5 mouse forelimb. beta-Gal(Pitx2) and Lbx1(EGFP) are co-localized in the flexor and extensor muscle groups. (**C**) FACS analysis of sorted Lbx1^EGFP/+^ cells. The automated multiwell plating function on the MoFlo was used to test a variety of substrate and media at systematically controlled plating densities. Cells were sorted at a rate of 10,000 cells/sec with a purity of 95–99+%, depending on the stringency of gating. Cell number (Y axis, log scale) vs. florescence intensity (X axis, FL1) plot. The “a” peak represented EGFP^−^ cell population, and the “b” peak represented the GFP^+^ cell population. The GFP^+^ population represents 5–7% of the total limb bud cellular pool. (**D**) RNA samples were quantified and ran on an Agilent Bioanalyser 2100 to assess RNA quality prior to microarray analysis. (**E**) Comparison of expression of total RNA from HET (y axis) vs. WT (y axis). Each dot in both axes represents relative RNA expression levels for an individual gene in WT vs. HET respectively. If a dot is perfectly located in the diagonal line, then the relative gene expression level for the representing gene exhibits no difference within HET and WT. (**F**) Comparison of expression of total RNA from MUT (y axis) vs. WT (x axis). Each dot in both axes represents relative RNA expression levels for an individual gene in MUT vs. WT respectively. (**G**) Comparison of expression of total RNA from MUT (y axis) vs. HET (x axis). Each dot in both axes represents relative RNA expression levels for an individual gene in MUT vs. HET respectively. Pitx2 expression levels were indicated by arrow. Pitx2 was strongly down regulated in the Pitx2 mutants. Comparison of expression of total RNA of genes from Table 1 of HET (y axis) vs. WT (x axis) (**H**), MUT (y axis) vs. WT (x axis) (**I**), MUT (y axis) vs. HET (x axis) (**J**).

To better understand the Pitx2 dependent mechanisms involved in muscle development, identification of Pitx2 target genes in forelimb muscle was initiated. During mouse embryonic development *Pax3* is expressed in the dermomyotome, whereas *Lbx1* is coexpressed with *Pax3* specifically in migratory hypaxial muscle precursors that undergo long-range migration to the limb buds and diaphragm [4], [6], [9]. We isolated the *Lbx1* population from forelimb tissue at E12.5 using Lbx1^EGFP^ mouse line. The Lbx1^EGFP^ mouse line [6] provides a robust system for developing genome-wide analyses of epistatic interactions in mammalian embryos. At E12.5, muscle progenitors in the limb have been segregated into distinct populations that mark the developing muscle anlagen. Lbx1^+^ marks and regulates MMP forelimb cells [6] (Fig 2A). The Lbx1 fluorescent cells from E12.5 embryos are also expressing Pitx2 [19]. The ratio of green to white cells accurately reflected the EGFP expression observed by immunohistochemistry (Fig 2B). Thus, fluorescence activated cell sorting (FACS) was used to purify the EGFP^+^ (G) and EGFP^−^ (W) cells from pools of 3–4 sets of forelimbs of MUT, HET and WT mice at E12.5 (Fig 2C). Total RNA from three biological replicates of each of the four conditions, HET green (hG), HET white (hW), MUT green (mG) and MUT white (mW), was used to probe Affymetrix Mouse 430 arrays (Fig 2D). Data from all twelve arrays were normalized using GC robust multi-array averaging in GeneSpring software (Fig 2E–J). The analysis focused on probe sets corresponding to genes involved in cell adhesion and motility. These annotated genes used in this analysis include: 18 adhesion, 9 microtubule, 9 cytoskeleton or cytoskeleton binding proteins and 2 signaling related functions. These 38 genes were collectively monitored by 102 probe sets. Genes with the greatest fold change in expression are involved in actin cytoskeleton, microtubule dynamics, cellular adhesion and contraction, extracellular matrix and signaling (Table 1).

**Table 1:** Pitx2 Target Genes In Forelimb Migratory Muscle Progenitor Cell Lineage.

Name	Transcript	Gene	WT	Het	Mut	Δ Fold	Function	Bibliography
**Actin Related Genes**								
1,2Actin alpha1	NM_009606	**Acta1**	1449±107	1544±349	2486±230	1.8	monomer	[66]
2Enabled Homolog (*Drosophila*)	NM_001008493	**Enah**	133±8	152±1	194±111	1.5	organization	[67]
2Actin γ2	NM_009610	**Actg2**	96±23	113±14	129±27	1.3	monomer	[68]
1,2Palladin	NM_001166108	**Palld**	294±21	352±23	376±113	1.3	organization	[69]
2Actinin alpha3	NM_01104	**Actn3**	1123±256	1017±88	650±17	−1.7	binding	[70]
1Cysteine and Glycine-rich Protein 1	NM_001193570	**Csrp1**	819±174	820±52	526±94	−1.6	dynamics	[71]
Protein Tyrosine Phosphatase	NM_001109992	**Ptpn11**	119±7	106±16	87±16	−1.4	dynamics	[72]
Tropomyosin 3	NM_001043351	**Tpm3**	192±43	196±18	155±29	−1.2	dynamics	[73]
Adenylate Cyclase-associated Protein 1	NM_001105530	**Cap1**	1253±274	1257±293	1031±338	−1.2	stability	[74]
**Microtubule Related Genes**								
1Microtubule-associated Protein Tau	NM_001123066	**Mapt**	44±3	51±13	256±144	5.8	dynamics	[75]
1Stathmin-like 2	NM_007029	**Stmn2**	32±14	26±2	131±32	4.0	dynamics	[76]
1Stathmin-like 3	NM_009133	**Stmn3**	71±2	62±21	236±106	3.3	dynamics	[76]
Shroom Family Member 3	NM_001077595	**Shroom3**	396±54	438±39	501±61	1.3	dynamics	[77]
2Tubulin beta4	NM_009451	**Tubb4**	130±56	141±84	165±29	1.3	monomer	[78]
1,2Tubulin beta3	NM_023279.2	**Tubb3**	170±61	113±11	216±93	1.3	monomer	[79]
1,2Tubulin beta 2b	NM_023716	**Tubb2b**	2216±312	2504±198	2766±426	1.2	monomer	[80]
1,2Tubulin beta5	NM_011655	**Tubb5**	2079±91	1670±66	1460±45	−1.4	monomer	[78]
Dynactin 4	NM_026302	**Dctn4**	129±13	122±31	94±45	−1.4	dynamics	[81]
**Adhesion Related Genes**								
1Myozenin 2	NM_021503	**Myoz2**	189±11	211±35	319±69	1.7	adhesion	[82]
1,2Integrin alpha4	NM_010576	**Itga4**	1985±261	2180±427	2920±663	1.5	adhesion	[83]
1,2A disintegrin-like and metallopeptidase with thrombospondin type 1 motif 9	NM_175314	**Adamts9**	169±92	167 ±32	239±63	1.4	adhesion	[84]
1Cysteine Rich Protein 61	NM_010516	**Cyr61**	521±22	542±6	732±183	1.4	adhesion	[85]
1Fibulin 2	NM_001081437	**Fbln2**	372±167	357±23	467±16	1.4	adhesion	[86]
SPARC Related Modular Ca-Binding 1	NM_001146217	**Smoc1**	261±27	282±40	347±40	1.3	adhesion	[87]
1Spectrin beta2	NM_009260	**Spnb2**	123±1	147±11	162±67	1.3	adhesion	[88]
1FAT Tumor Suppressor Homolog 3	NM_001080814	**Fat3**	441±55	466±8	578±72	1.3	adhesion	[89]
2Ring finger protein 165	NM_001164504.1	**Rnf165**	124±20	152±42	160±16	1.3	adhesion	[90]
1,2Syndecan 4	NM_011521	**Sdc4**	188±15	200±5	127±29	−1.5	ECM reorganization	[91]
1,2Syndecan 2	NM_008304	**Sdc2**	360±42	404±61	251±37	−1.4	ECM reorganization	[92]
2A disintegrin-like and metallopeptidase domain 19	NM_175506	**Adam19**	250±24	222±14	192±0	−1.3	adhesion	[93]
1Connective Tissue Growth Factor	NM_010217	**Ctgf**	158±44	198±87	104±22	−1.3	adhesion	[94]
Sphingosine-1 Phosphate Phosphatase 1	NM_030750	**Sgpp1**	169±43	145±6	128±33	−1.3	adhesion	[95]
1Junction Adhesion Molecule 3	NM_023277	**Jam3**	1258±137	1197±55	959±38	−1.3	adhesion	[96]
LIM Domain Only 7	NM_201529	**Lmo7**	180±96	158±56	141±57	−1.3	adhesion	[97]
1Periostin	NM_001198765	**Postn**	2110±186	1722±28	1625±77	−1.3	adhesion	[98]
2Tetraspanin 33	NM_146173	**Tspan33**	367±133	375±30	300±23	−1.2	adhesion	[99]
**Signaling Related Genes**								
1,2Protein kinase, cAMP dependent regulatory, type II beta	NM_011158	**Prkar2b**	118±9	137 ±58	185±15	1.6	cAMP regulation	[100]
1,23-phsphoinositide dependent protein kinase 1	NM_001080773	**Pdpk1**	632±127	356±34	293±114	−2.2	protein kinase activity	[101]

1 indicates one of the three array sets was inconsistent with the other two, expression means and standard deviation was calculated using only the two consistent arrays.

2 members of the adhesome.

### Cytoskeletal Defects in Myogenic Cells in Pitx2 Mutants

**Figure 3:**
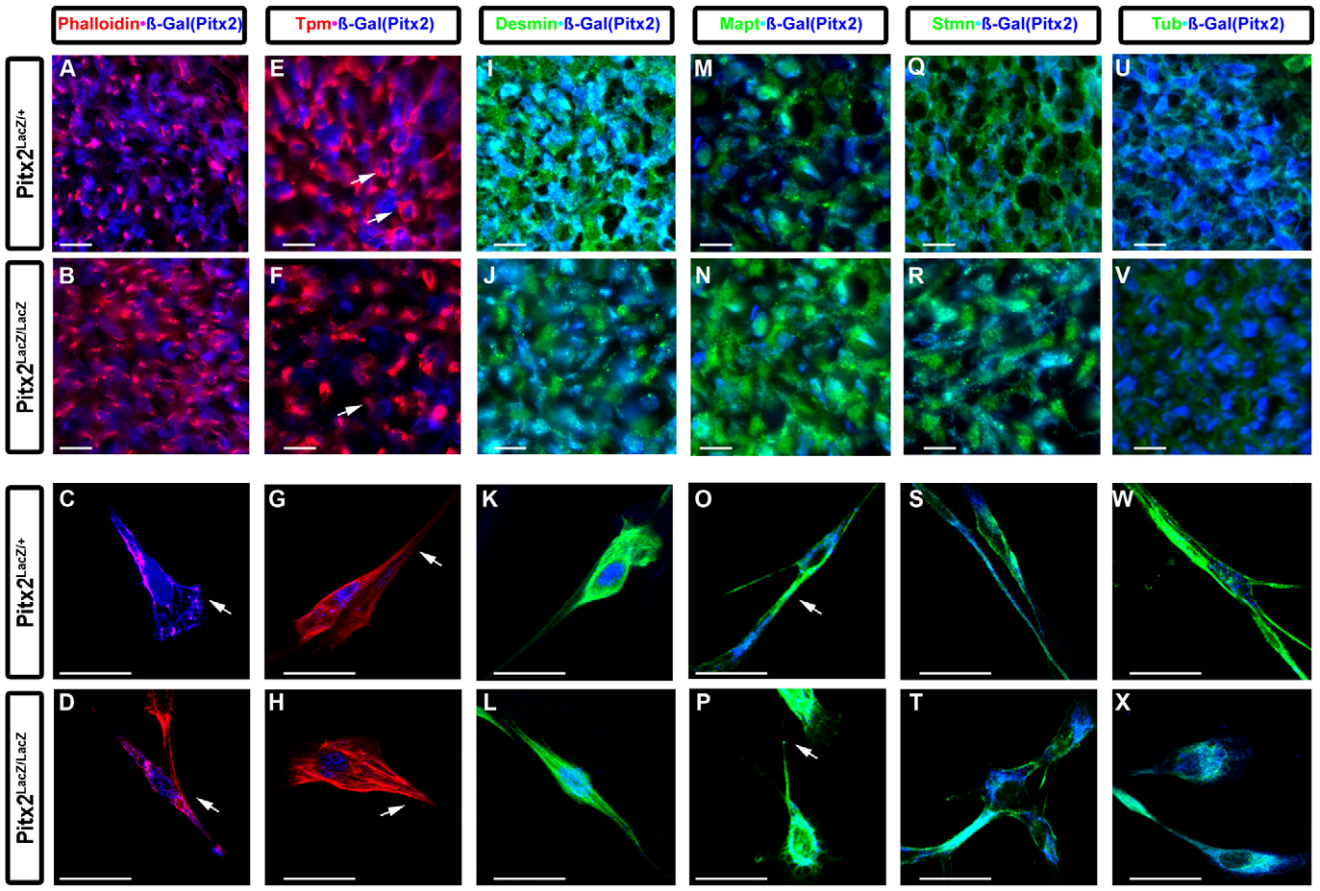
Increased Actin Bundling and Presence of Tau and Stathmin in Pitx2 Mutant Myogenic Cells. Immunostaining for Phalloidin (F-Actin) and beta-Gal(Pitx2) (**A–D**), tropomyosin and beta-Gal(Pitx2) (**E–H**) desmin (Intermediate Filament) and beta-Gal(Pitx2) (**I–L**) on Pitx2^LacZ/+^, Mapt (Tau) and beta-Gal(Pitx2) (**M–P**), stathmin (stmn) and beta-Gal(Pitx2) (**Q–T**), and tubulin (tub) and beta-Gal(Pitx2) (**U–X**) on Pitx2^LacZ/+^. The F-actin, tropomyosin and desmin labeled fibers in cells in the MUT forelimbs were not aligned and cluster together as in the HET. MUT myogenic cells failed to develop protrusions, connect and align to each other. Mapt and stmn were highly expressed in forelimb tissue and myogenic primary cultured cells. Mapt, stmn and tub expression levels were increased in both tissue and primary myogenic cells cultures. Arrows denote points of interest between genotypes. White bar denotes 50 micrometer.

The expression of numerous genes encoding for cytoskeletal components or proteins regulating the dynamic assembly and disassembly of cytoskeletal components were altered in Pitx2 MUT myogenic cells. To investigate if these alterations in gene expression resulted in cytoskeletal defects immunohistochemistry on E12.5 limbs and on primary cultured limb myogenic cells were performed in series of double labeling experiments. Phalloidin was used to visualize the actin filaments (F-actin) and beta-Gal to visualize the expression of Pitx2^LaZ^. Special care was given to positioning both HET and MUT forelimbs for cross sectioning of the forelimbs. Actin filaments were equally distributed represented with a round shape in the HET forelimb muscle tissue (Fig 3A) while they were clustered together forming long fibers in the MUT (Fig 3B). Cultured myogenic cells were characterized with a smooth flat shape with several protrusions with filaments at the border of the cell (Fig 3C, arrow) while MUT cells were smaller, less developed with increased actin filaments along their body (Fig 3D, arrow). The muscle specific actin binding protein tropomyosin (Tpm) had very similar expression pattern (Fig 3E, F). Forelimb muscle sections from HET tissue indicated that cells were surrounded by orderly Tpm fibers (3E, arrows), while in MUT cells were surrounded by thicker denser looking fibers with Tpm forming a ring around them (3F, arrow). Cultured HET cells had an elongated shape with thin smooth fibers throughout the entire body and able to come together for further fusion (Fig 3G, arrow), conversely, to the MUT cells which exhibited a more round appearance with shorter thicker fibers (Fig 3H, arrow). The muscle specific intermediate filament protein desmin was not significantly mis-regulated in the gene expression arrays. However, its distribution did change in the forelimb tissue of the MUT, with desmin positive muscle cells not tightly connected and positioned without a distinct anatomical formation (Fig 3I, J). Cultured HET cells were more finely fibered and had an elongated shape (Fig 3K). In contrast, the MUT cells had shorter and thicker filaments (Fig 3L).

Similar approach was used to investigate if genes that encode for microtubules components, organization, or regulate dynamics resulted in defects. Microtubule Associated protein Tau (Microtubules) had a lighter more diffuse staining throughout the cell body in HET (Fig 3M) compared to the strong dense levels in MUT (Fig 3N). Myogenic HET isolated cultured cells had elongated cell shape (Fig 3O, arrow), in contrast to the MUT cells with a smaller rounder appearance and increased Tau levels (Fig 3P, arrow). Stathmin 2 (stmn) immunostaining was light and diffused in the cell body of HET muscle forelimb cells (Fig 3Q) while it was highly expressed in the segregated MUT cell bodies in a disorganized manner forming a web like structure (Fig 3R). Similarly, stmn expression was elevated in the body of the MUT stumpy looking cultured cells (Fig 3T). Tubulin expression was highly detected in MUT tissue (Fig 3V) and isolated cells (Fig 3X). Myogenic MUT cells maintained round separated and failed to elongate and contact with each other to form multinucleated myofibers (Fig 3D, H, L, P, T, X). Thus, Pitx2 might act as a balance factor for (1) the formation of cytoskeleton by regulating the molecular ratio of thin and intermediate filaments and (2) the cell motion by regulating microtubules.

### Defected Focal Adhesions in Myogenic Cells in Pitx2 Mutants

The directional migration of cells is initiated by extracellular cues. Initiation of migration occurs by polarizing and extending a protrusion, containing the broad lamellapodia and spiky filopodia, of the cell membrane towards the cue. Both of these structures are driven by polymerization of actin filaments, which then stabilized by adhering the actin cytoskeleton to the ECM. Signals from the newly formed, more stable and mature adhesions influence cytoskeletal organization, which in turn influences the formation and disassembly of the adhesions. This feedback loop coordinates spatial dynamics and mechanical stresses that lead to directional cell movement. Cells express cell surface adhesion receptors integrins that anchoring them to extracellular matrices and alter their function by activating intracellular signaling pathways after ligand binding. The integrin-actin linkage is mediated by several proteins. Talin is an actin-binding protein that binds integrin tails and transitions integrin to an active state.

**Figure 4:**
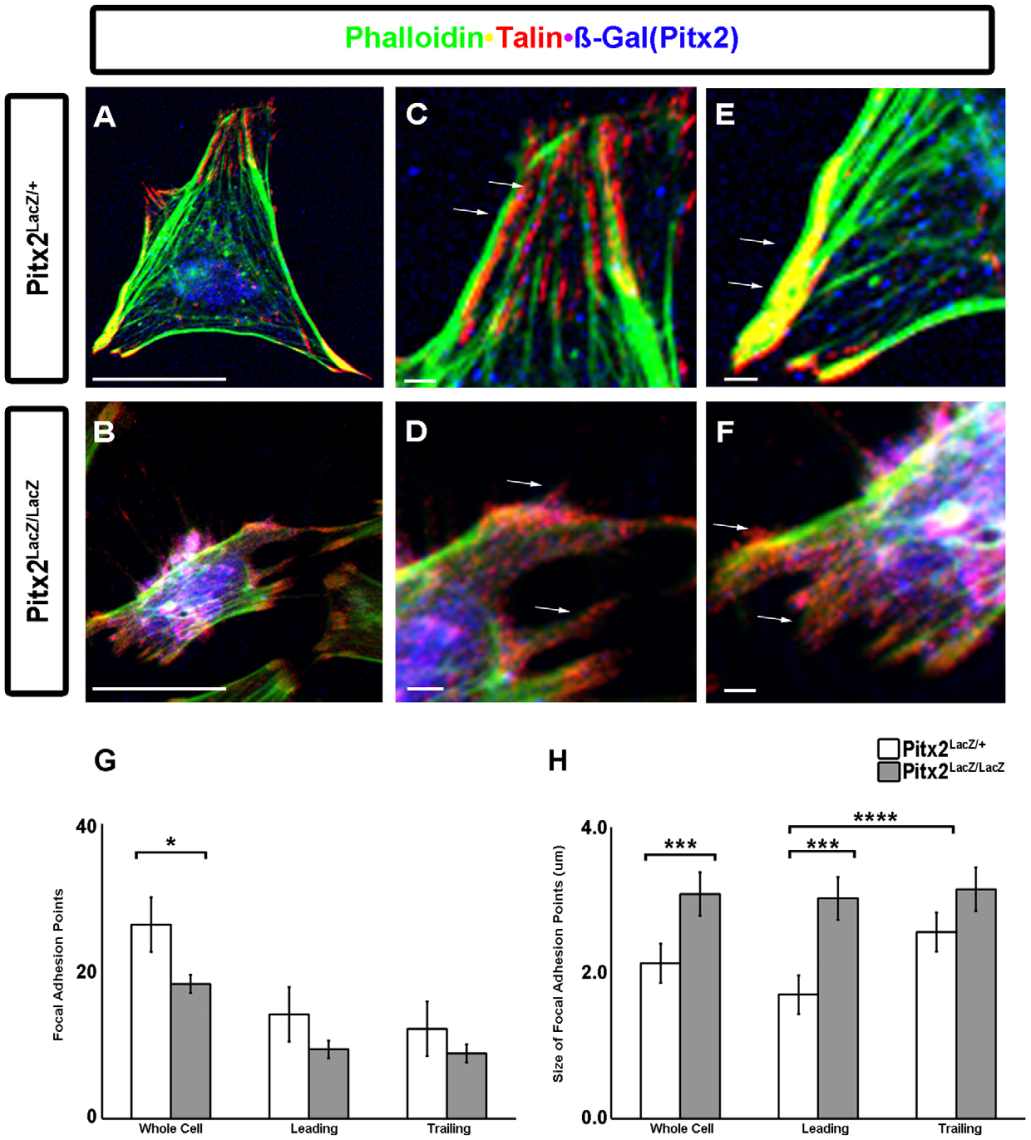
Altered Focal Adhesion in Pitx2 Mutant Myogenic Cells. Muscle progenitor cells were isolated from E12.5 forelimb tissue of Pitx2^LacZ/+^ (**A, C, E**) and Pitx2^LacZ/LacZ^ (**B, D, F**) mice. Cells were stained with alexa 488 phalloidin, (F-actin), talin (focal adhesions) and beta-Gal(Pitx2) (**A–F**). (**G**) Myogenic cells had a mean ± standard error of the mean (SEM) of 26±4 (total = 475) for HET and 18±1 (total = 330) for MUT of focal adhesions per cell (n = 18). This difference in mean focal adhesion number was determined statistically significant using a two-tailed unpaired t-test (p = 0.0464). The distribution of the number focal adhesions between the leading and trailing edges of the muscle progenitor cells was not affected; the leading edges had an mean ± SEM of 14±2 (total  = 255) for HET and 9±1 (total  = 170) for MUT cells, while at the trailing edges had means ± SEM of 12±2 (total  = 220) for HET and 9±1 (total  = 160) for MUT cells. Neither of the differences of focal adhesion number at leading or trailing edges between HET and MUT cells were determined statistically significant using two tailed unpaired t-test (p = 0.05 and p = 0.0848, respectively). While differences between the leading and trailing edges within HET or MUT cells were also determined not statistically significant using two tailed paired t-test (p = 0.219 and p = 0.355). (**H**) The size of focal adhesions had an mean size ± SEM of 2.14±0.27 micrometer in HET and 3.09±0.3 micrometer in MUT cells. This difference in focal adhesion size was determined to be very statically significant using two-tailed unpaired t-test (p = 0.0013). The mean size ± SEM of focal adhesions at the leading edge was 1.70±0.24 micrometer for HET and 3.03±0.3 micrometer for MUT, while at the trailing edge the mean size ± SEM was 2.57±0.26 micrometer for HET and 3.15±0.3 micrometer for MUT. The mean focal adhesion size at the leading edge between HET and MUT cells were determined to be very statistically significantly different using unpaired two tailed t-test (p-value  = 0.0016). When comparing leading and trailing focal adhesion size within HET cells there is a statistically significant difference in mean size two-tailed paired t-test (p-value  = 0.001). Comparing mean focal adhesion size between leading and trailing edges within MUT cells showed no statistically significant difference using two tailed paired t-test (p  = 0.1325). White bar denotes 50 micrometer.

The expression of numerous genes involved in adhesion and in actin cytoskeleton has been altered in the Pitx2 mutants, suggesting that the formation of nascent adhesions in this cell population should be malformed. Forelimb cultured myogenic cells from HET and MUT mice were subject to triple labeling immunostaining for beta-Gal (Pitx2), talin to detect the focal adhesions and Phalloidin to detect the F-actin stress fibers with (Fig 4). Talin and actin were coexpressed along the cell body, identifying the focal points (Fig 4 A, C, E). Talin and actin coexpression was weakly detected in very few locations of the cells with small cytoplasm, suggesting that the focal points were limited in the MUT cells (Fig 4 B, D, F). As cells migrate through their environment the cytoskeleton stresses and contracts forming a leading and a trailing edge respectively. The nucleus moves towards the trailing edge allowing space for the cell to extend its cytoplasm (Fig 4A), while in the MUT cells the nucleus occupied the most of the body (Fig 4B). Focal adhesion points stained for both talin and Phalloidin were identified and counted in both HET and MUT cells. HET cells characterized with distinct trailing stress fibers and a smooth leading (Fig 4C) and trailing edge (Fig 4E) and similar number of focal adhesion points (Fig 4G). In MUT cells trailing and leading edges were not distinct with reduced number of focal adhesion points by 36% in leading and 25% in trailing edge (Fig 4G). However, the size of focal adhesion points was increased in MUT cells by 25% compared to HET cells (Fig 4H).

The observed phenotype of the myogenic cultured cells (Fig 3 and 4) was in accord with the phenotype observed in limb muscle groups (Fig 1). The clumpy, disorganized and truncated cells resulted to the formation of a dense and sort in length muscle. The leading edge, the more dynamic end of the cell, changes rapidly in order for the cell to sample its environment. Disruption of microtubules using pharmacological agents results in loss of cell polarity, increase in focal adhesion size, and formation of actin stress fibers [27]. At the leading edge of the cell, formation of many nascent adhesions to anchor the cell to the substratum occurs. Nascent adhesions are small (<1.0 micrometer) transient structures. These nascent adhesions rapidly assemble and disassemble allowing for sampling of the environment prior to formation of more stable or mature contacts. The maturation of focal adhesions are accompanied by an increase in size (2–10 micrometer) and the formation of actin stress fibers that terminate at the focal adhesion, presumable to allow for contraction of the cell body to propel the cell forward. Disruption of the microtubule dynamics leads to the formation of larger focal adhesions, loss of cell polarity, and increased formation of actin stress fibers [27]. Thus, we suggest that adhesion irregularities of myogenic cells delay their ability to move fast and populate their muscle anlagen.

### Impaired Motility of Myogenic Cells in Pitx2 Mutants

**Figure 5:**
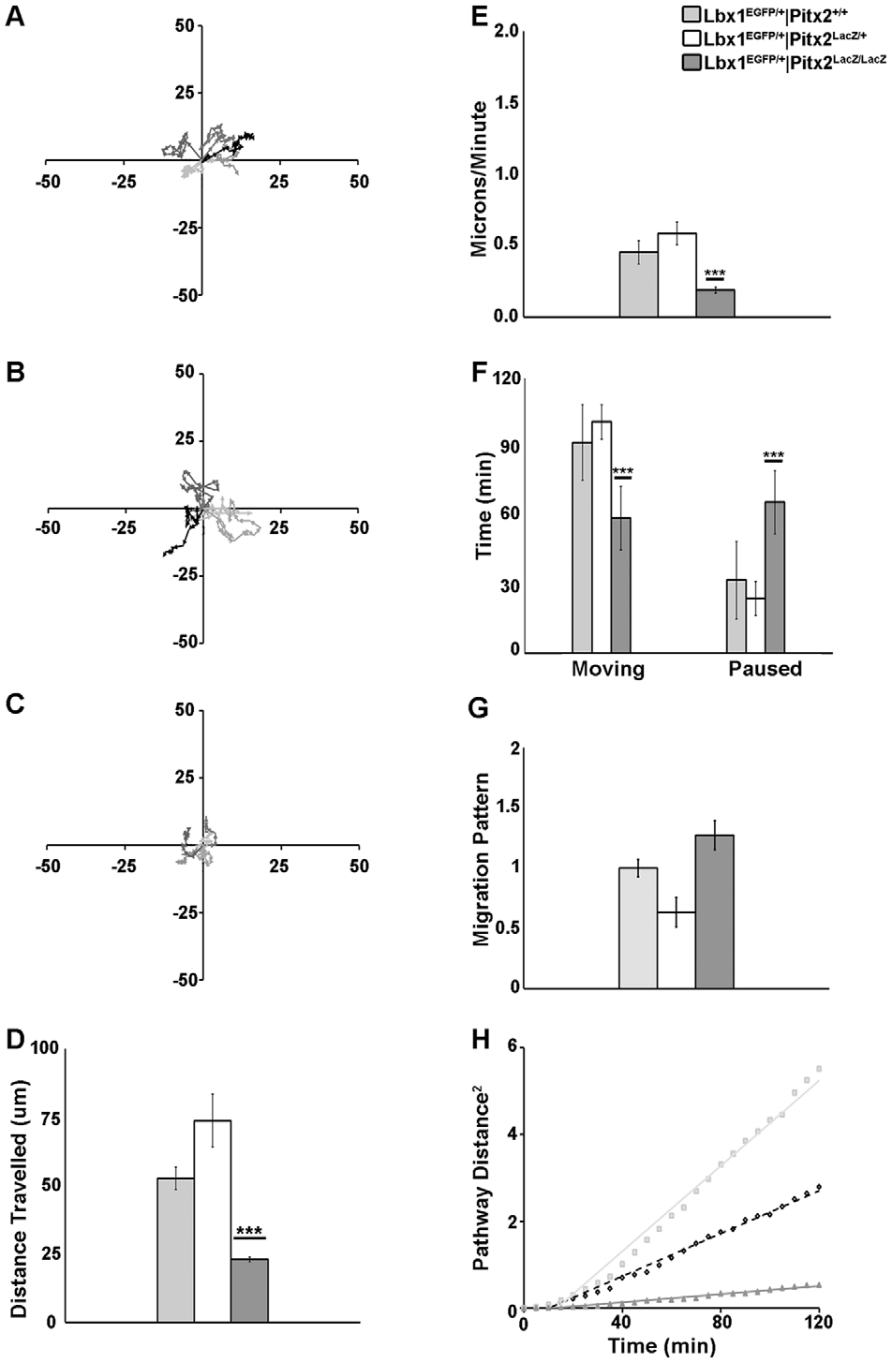
Motility Defects in Lbx1^+^ Myogenic Cells in Pitx2 Mutants. Live cell tracking assay of migratory muscle progenitors (n = 5) isolated from forelimb tissue of E12.5 Lbx1^EGFP/+^|Pitx2^+/+^ (WT), Lbx1^EGFP/+^|Pitx2^LacZ/+^ (HET), or Lbx1^EGFP/+^|Pitx2^LacZ/LacZ^ (MUT) embryos. Migration pathway of migratory muscle progenitors over a 2 hour period for WT (**A**), HET (**B**), MUT (**C**). **(D)** Mean total distance travelled of WT (53±4 micrometer), HET (74±10 micrometer), and MUT (23±1 micrometer). (**E**) Mean velocity of movement of WT (0.5±0.1 micrometer/min), HET (0.6±0.1 micrometer/min) and MUT (0.2±0.02 micrometer/min). (**F**) Mean time spent moving vs. paused for WT moving (92±16.4 min) and paused (32±16.8 min), HET moving (101±7.4 min) and paused (24±7.4 min) and MUT moving (59±14 min) and paused (66±14 min). Using Dunnett's ANOVA test setting WT as control, the MUT MMPs were found to be significantly different in distance travelled, velocity, and time moving vs. paused. Following Dunnett's ANOVA an unpaired T-test between WT and MT determined significance values for distance traveled (p = 0.0001), velocity (p = 0.0001), time moving (p = 0.0089) and time paused (p = 0.0082). (**G**) Quantitation of persistent migratory directionality. Relative ratios of direct distance from start point to end point (D) divided by the total pathway distance traveled (T), ratios expressed as relative to WT (value set = 1.0). The MMPs from HET had a ratio of 63% and MUT MMPs had a ratio of 127%. (**H**) The mean square displacement of total pathway distance traveled (T^2^) measured every 20 min. The x-intercept HET (diamonds, black dotted line) and WT (light grey squares, solid light grey line) cells were as close to the origin as the intercept for MUT (dark grey triangles, solid dark grey line), indicating that cells from all genotypes exhibit similar migration behaviors.

The hypocellularity and distortion of forelimb muscle groups observed in Pitx2 MUT mice (Fig. 1) might be the result of impaired migration of MMP cells to the distal forelimb. Live imaging of primary cultures of E12.5 forelimb MMP cells from Lbx1^EGFP/+^|Pitx2^+/+^ (WT), Lbx1^EGFP/+^|Pitx2^LacZ/+^ (HET) and Lbx1^EGFP/+^|Pitx2^LacZ/LacZ^ (MUT) (Fig 5) and proliferating MP cells from Pax3^Cre/+^|ROSA^EGFP^|Pitx2^+/+^ (WT), Pax3^Cre/+^|ROSA^EGFP^|Pitx2^LacZ/+^ (HET) and Pax3^Cre/+^|ROSA^EGFP^|Pitx2^LacZ/LacZ^ (MUT) (Fig S2) was performed. Individual cells were visualized by EGFP expression and changes in position were recorded every 5 min for a period of 2 hrs. MMP WT cells were migrated in a random fashion with cells frequently moving and several changes in direction (Fig 5A). MMP HET cells migrated in a similar fashion with cells frequently moving but with fewer changes in direction (Fig 5B). MMP MUT cells migrated much differently with cells spending more time paused and with fewer changes in direction (Fig 5C). MUT cells traveled half the distance of WT and almost 1/3 of the HET (Fig 5D). Velocity was also decrease in MMP MUT (0.2±0.02 micrometer/min) compared to HET (0.6±0.1 micrometer/min) and WT (0.5±0.1 micrometer/min cells), (Fig 5E). Migratory behavior of MMP cells was also altered. MUT cells spent more time paused (66±14 min) than moving (59±14 min), while WT (92±16 min moving, 32±17 min paused) and HET (101±7 min moving, 24±7 min paused) cells were moving more and spent only 1/3 or 1/4 of their time paused respectively (Fig 5F). All differences were determined statistically significant using a Dunnett's ANOVA using WT as the control group, followed by an unpaired T-test between WT and MT determined significance values for distance traveled (p = 0.0001), velocity (p = 0.0001), time moving (p = 0.0089) and time paused (p = 0.0082). To quantify differences in migration patterns, the ratios of the shortest direct distance from the starting point of each recording to the end point (D), to the total track distance of the cell (T) was compared [28]. The ratio D/T to a value of 1 using data collected from MMP WT cells was normalized. MMP MUT cells showed an increased ratio of 127%, while HET showed reduced ratio of 63%, compared to WT cells (Fig 5G). The random vs. directional cell motility, was measured by a mean square displacement assay [29]. The mean square displacement of total pathway distance traveled (T^2^) measured every 20 min was calculated and plotted against time. If movement is purely random, the linear regression line would pass through the origin. The x-intercept for HET cells was as close to the origin, as the intercept for MUT cells exhibited migration behaviors, (Fig 5H).

Similar analysis was performed in proliferating MP cells (Fig S2). WT and HET cells migrated in a random fashion similar to MMP cells with the exception that MP cells tended to persist in a single direction longer before changing.

Data from these studies suggest that myogenic cells take longer time to populate the limb anlagen in Pitx2 mutants due to their random movement and reduced velocity. As they proliferate they continue to migrate in a slower pace. This delay to reach their final destination does not follow the general growth limb program and the forming muscle is distorted and disorganized in the Pitx2 mutants.

## Discussion

**Figure 6:**
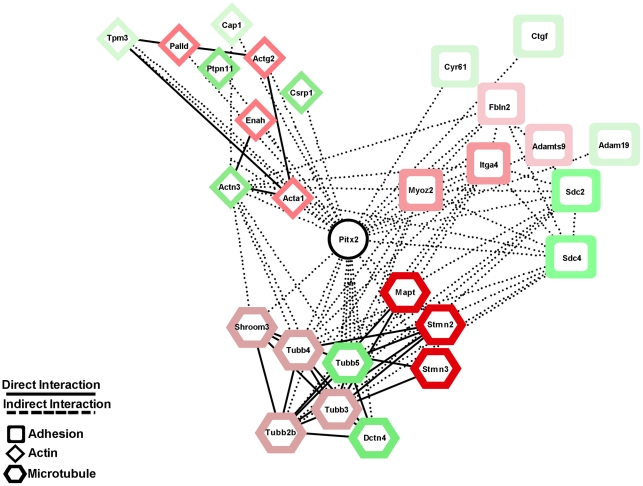
Pitx2-Mediated Myogenic Cell Gene Network During Filling Limb Muscle Anlagen. The network displays ten genes with the greatest fold change from each of the three groups; actin (diamonds), microtubule (octagons) and adhesion (squares) related genes. Color intensity is representative of reported fold change based on their gene expression analysis (Table 1), with darker color representing larger fold change. Connections between the nodes are displayed as solid lines for direct interactions and dashed lines for possible interactions.

In this report we identified a cadre of Pitx2 target genes that function as components of or act in the assembly, organization and regulation of the cytoskeleton in forelimb myogenic cells (Table 1, Fig 6). In order to migrate efficiently the cell must respond to both intracellular and extracellular cues that reorganize the cytoskeleton. This constant reorganization influences the cell morphology and ultimately cell fate. The leading edge of the migrating cell is dominated by actin based structures lamellapodia and filopodia. Actin based cell motility is highly dynamic, conserved across eukaryotes and a fundamental process driving tissue development [30]. A network of proteins link the internal cytoskeleton of the cell to the external environment through adhesion molecules, allowing for the generation of force needed for cell movement [31].

The actin isoform actin alpha 1 (*Acta1*) is expressed in skeletal muscle tissue [32] and its expression was inhibited by Pitx2 (Table 1, Fig 6). In the initial stages of elongation *Acta1* expression levels increase in multinucleated myotubes while it localizes with the sarcomeric thin filaments [33]. Overexpression of *Acta1* reduces the ability of cell spreading and elicit the expression of a subset of muscle differentiation genes in the absence of normal muscle differentiation without withdrawal from the cell cycle [34]. The muscle specific alpha-actinin-3 (*Actn3*) acts as scaffolding protein that cross-links actin stress fibers to focal adhesion proteins providing anchor points, regulates the activity of a number of cell surface receptors, and acts as a scaffolding protein to connect the cytoskeleton to signaling pathways [14]. The cysteine and glycine-rich proteins (CRPs) are a subset of LIM domain protein family. *Csrp1* is ubiquitously expressed in striated and smooth muscle [35] and its expression, was promoted by Pitx2 (Table 1). *Csrp1* binds to alpha-actinin, which binds and bundles actin filaments into stress fibers and is responsible for maintaining proper tissue homeostasis [36]. The isoform Pitx2a has been previously shown that implicated in regulating actin cytoskeleton dynamics indirectly through Rho GTPases in HeLa cells [37]. However, in our microarrays in forelimb migratory muscle precursor cells no mis-regulation of genes involved in Rho GTPase signaling was detected.

A downstream target of Rho GTPase signaling pathway is the cyclic-nucleotide-dependent kinase PKA which act to phosphorylate Ena/VASP proteins and promote their dissociation from the focal adhesion terminating actin fiber formation and negatively regulating lamellapodia formation [38]. The mammalian homolog of *Drosophila* enabled (*Enah*) *Mena* is a member of the enabled/vasodilator-stimulated phosphoprotein (*Ena/VASP*) family of actin regulatory proteins that act as critical regulators of actin assembly and cell motility with increased levels in heart failure [39], [40]. Ena/VASP proteins bind to the focal adhesion proteins zyxin and vinculin, and to the actin nucleation protein profilin where it prevents capping and branching of the growing actin filament by the Arp2/3 complex. This complex promotes growth and formation of actin stress fibers allowing for protrusion of the cell membrane forming lamellapodia. The Ena/VASP proteins are also substrates for the cyclic-nucleotide-dependent kinases PKA and PKG, which act to phosphorylate Ena/VASP proteins and promote their dissociation from the focal adhesion terminating actin fiber formation and negatively regulating lamellapodia formation [41]. Pitx2 acted as an inhibitor of Enah in myogenic cells (Table 1, Fig 6).

Another gene family that was affected in Pitx2 MUT myogenic cells was the microtubule related gene family (Table 1). Microtubules are an active constituent during cell migration. They are organized with the growing end toward the leading edge that extends to the base of the lamellipodium. The constant growth and shrinkage (dynamic instability) of the microtubules is required for cell migration. Pharmacological agents that either promote stabilization or depolymerization microtubules result in reduction in migration [42]. Tau binds to the outside surface of the microtubule and stabilizes the microtubule promoting growth [43]. Rho GTPase signaling influences microtubule dynamics through activation of Cdc42 and Rac. The activation of these two proteins leads to the phosphorylation of the microtubule destabilization protein stathmin [44]. The phosphorylation of stathmin leads to the inhibition of its ability to form ternary complexes with tubulin dimers preventing their incorporation into growing microtubules and promoting shrinkage of the microtubules [45]. Stathmins form ternary complexes with tubulin dimers preventing their incorporation into growing microtubules and promoting shrinkage of the microtubules [45]. During migration microtubules undergo dynamic instability to explore the intracellular environment. Growing microtubules are stabilized to focal adhesion complexes [38]. During myogenic differentiation the transition from myoblast to myotube is accompanied by extensive changes in morphology and reorganization of the cytoskeleton [46]. During myotubes formation the individual myoblasts' eliminate their microtubule organizing centers (MTOC) and align themselves into stabilized linear arrays along the multinucleated myotubes.

The expression profile of adhesion related genes were altered in the Pitx2 MUT forelimb myogenic cells (Table 1). Myozenin 2 (*Myoz2*) binds to alpha-actinin and γ-filamin and colocalized with alpha-actinin and gamma-filamin along the Z-Disc of striated muscle where it organizes and spaces the actin thin filament [47].

Integrins are transmembrane receptors that mediate attachment between the cell and the surrounding environment via cell-cell contacts or to the extracellular matrix (ECM). They provide anchoring points for the cytoskeleton and transduce environmental information to inside the cell thereby regulating cell shape, motility, differentiation, and the cell cycle [48]. Integrins consist of two transmembrane subunits, the alpha controls selective binding to substrate, and the beta controls signal transduction into the cell [49]. Integrins are clustered at focal adhesions along with other phosphorylated adhesion proteins: paxillin, talin and focal adhesion kinase (FAK) which initiate signaling cascades that lead to activation of protein kinase C (PKC) that promote muscle cell survival, spreading and migration [50], [51].

Syndecans are type 1 transmembrane heparin sulfate proteoglycans (HSPGs) that contain a short cytoplasmic domain, a transmembrane domain, and a long intracellular domain [52]. *Sdc4* is colocalized to focal adhesions containing talin, vinculin, alpha-actinin, paxillin, and FAK while its overexpression results in excessive focal adhesion formation and reduced cell migration [53]. After focal adhesion have formed *Sdc4* modulates focal adhesion strength through recruitment of the GTP•RhoA protein, which acts to strengthen and cap focal adhesions preventing further actin stress fiber formation, and Rac1, which acts to weaken focal adhesions allowing for increased stress fiber formation and protrusion of lamellapodia [54], [55].

The protein kinase cAMP regulatory subunit II beta (*Prkar2b*) is a key enzyme for the regulation of the protein kinase A (PKA) was also regulated by Pitx2. PKA is a positive and negative regulator of cell migration and is spatially enriched at the leading edge of the cell [56]. At the leading edge PKA is required for the activation of Rac and Cdc42 proteins promoting actin filament assembly; conversely it inhibits the proteins Rho and PAK, as well as VASP proteins [57], [58].

The 3-phosphoinositide dependent protein kinase 1 (*Pdpk1*) is recognized as a master kinase of the cell required for the activation of many signaling pathways. The Pdpk1 protein is phosphorylated by 3-phosphoinositide kinase (PI3K) protein. Phosphatidylinositol (PI) signaling is complex and crucial for migration, in general signaling through PKA and the G-proteins (Rac, Rho and Cdc42) at the leading edge leads to increased PI levels due to activation of type 1 phosphatidylinositol kinases (PIPK1) resulting in an increase in actin polymerization [59]. At the trailing edge, the protein Calpain acts to begin disassembly of focal adhesions using PIs as a substrate to dissociate integrins from the cytoskeleton [60].

Pitx2 affects muscle specification in the jaw but appears to disrupt higher-order muscle assembly in virtually all skeletal muscles. In limb muscle, it does so without affecting muscle specification or cellular muscle differentiation. Despite all of this apparent normality, the muscle anlagen become oddly distorted and granular 1–2 days after being colonized, suggesting that Pitx2 function becomes critical between colonization and myofibril assembly. Our results indicate that anlagen volume and proliferative indices differ in mutant anlagen, and suggest that there is a defect in the mechanism that maintains correct progenitor pool size as muscles enlarge and engage in higher-order assembly. Many cytoskeletal proteins with established roles in cellular motility and adhesion in other systems have significantly altered expression levels in myogenic cells. Embryos use transcriptional network states during pattern formation to specify where and when muscle progenitor cells will form in the developing body plan (Fig 6). These muscle progenitor cells proliferate, express MRFs, and colonize particular regions of the body to form muscle anlagen. The anlagen assemble on a pre-pattern that is also set up by pattern formation processes. Once myoblasts arrive at their anlage they begin to pull out of the cell cycle and engage in higher-order assembly of muscle. Higher-order assembly is likely to happen in a similar way at all anatomical positions, but still needs to be understood at the molecular level to help understand how myopathies form and can be cured.

## Materials and Methods

### Mice

ICR Pitx2^LacZ/+^ mouse embryos (HET) [22], Lbx1^EGFP/+^ [6], Pax3^Cre/+^ [61] and Rosa^EGFP/+^ [62] were used. Pitx2^LacZ/+^ mice were bred with Pitx2^LacZ/+^, Lbx1^EGFP/+^ and Pax3^Cre/+^|Rosa^EGFP/+^ to generate Lbx1^EGFP/+^|Pitx2^LacZ/LacZ^, Lbx1^EGFP/+^|Pitx2^LacZ/+^, Lbx1^EGFP/+^|Pitx2^+/+^, Pax3^Cre/+^|Rosa^EGFP/+^|Pitx2^LacZ/LacZ^, Pax3^Cre/+^|Rosa^EGFP/+^|Pitx2^LacZ/+^, Pax3^Cre/+^|Rosa^EGFP/+^|Pitx2^+/+^ mice. Genomic DNA was extracted from tail and used for PCR genotyping [6], [22]. For cell flow sorting, embryos were rapidly genotyped under a fluorescent microscope to identify Lbx1 HET mice. To identify the Pitx2 genotypes, only Lbx1 HET embryos were subjected to X-gal staining.

### X-Gal Staining

Mouse embryos at E12.5 were washed with PBS and incubated with 1 mg/ml X-Gal in 2 mM MgCl_2_, 0.02% NP40, 5 mM K_3_F_4_(CN)_6_, 5 mM K_4_F_3_(CN)_6_ in PBS. For whole body staining embryos were incubated at 37°C O/N and for quick genotyping dissected heads were incubated for at least 0.5 h. Samples were washed with PBS and clarified with glycerol for analysis and photography.

### Flow-Sorting EGFP^+^ Forelimb Myogenic Cells

Synchronous Lbx1^EGFP/+^ containing litters were removed at E12.5 and rapidly genotyped under a fluorescent microscope to identify Lbx1^EGFP/+^ HET embryos and X-gal staining to identify Pitx2 MUT and HET. Limb buds and ventral body wall compartments between the caudal edge of the shoulder and lumbar region were dissected. For enzymatic dissociation, 40 limb buds were incubated in 1 ml of dissociation buffer (HBSS without HEPES [Hyclone; Rockford, IL USA], 1 mg/ml Type I Collagenase [Worthington Biochem; Lakewood, NJ USA]) for 3 min at 37°C. Large tissue was disrupted by 10 times repetitive pipetting through a 1 ml tip with an additional pipetting followed by 1 ml quench buffer (DMEM/F12 with 15 mM HEPES and 2.5 mM Glutamine [Hyclone; Rockford, IL USA], 25 microgram/ml BSA Fraction V [Sigma; St. Louis, MO USA], 0.5 M EDTA, 100 mM EGTA, 50 U/ml Pen/Strep [Cellgro; Manassas, VA USA], 0.25 microgram/ml Fungizone [Invitrogen; Carlsbad, CA USA]). Cells were filtered through 30 micrometer^2^ Nitex filter, centrifuged and flow sorted using MoFlo high-performance cell sorter [Dako Colorado Inc.; Carpinteria, CA USA].

### Propidium Iodide Staining and Cell Cycle Analysis

Flow sorted PAX3^CRE^|ROSA^EGFP^ cells were collected in 5 ml culture tubes containing PBS. These cells were centrifuged at 300×g for 5 min, supernatant was discarded and pellet resuspended in PBS. Cells were centrifuged and supernatant was discarded and the pellet resuspended in 0.5 ml PBS +0.1% Triton-X 100 in addition to 10 microliter of RNase A (10 microgram/ml; [Invitrogen; Carlsbad, CA USA]) and 10 microliter Propidium Iodide (1 mg/ml; [Sigma-Aldrich, St. Louis, MO USA]). Cells were incubated for 30 min at room temp prior to cell cycle using FC500 flow cytometer [Beckman Coulter; Brea, CA USA].

### Extraction of Total RNA

Flow sorted EGFP^+^ cells (green, G) and EGFP^−^ cells (white, W) were lysed with RLT buffer [Qiagen; Valencia, CA USA], (2×10^6^ cells/350 microliter). For extraction of total RNA RNAeasy Micro Kits [Qiagen; Valencia, CA USA] were used according to manufacture protocols.

### RNA Preparation and Microarray Analysis

Lbx1^EGFP^ forelimb cells were enriched from three pools of WT (Lbx1^EGFP^|Pitx2^+/+^), HET (Lbx1^EGFP^|Pitx2^LacZ/+^), and MUT (Lbx1^EGFP^|Pitx2^LacZ/LacZ^) embryos by cell sorting using MoFlo high-performance cell sorter [Dako Colorado Inc.; Carpinteria, CA USA] on basis of EGFP signal. Total RNA was prepared from forelimbs and probes prepared from these RNA were applied to nine Mouse Genome 430 2.0 microarrays [63]. The results from all nine arrays were normalized by RMA. The mean expression value obtained from three biological replicates was compared between genotypes. The data discussed in this publication have been deposited in NCBI's Gene Expression Omnibus [64] and are accessible through GEO Series accession number GSE31945 (http://www.ncbi.nlm.nih.gov/geo/query/acc.cgi?acc=GSE31945).

### Myogenic Cell Cultures

Forelimbs from E12.5 Pitx2^LacZ^ HET and MUT mice were dissected and dissociated into single cells in dissociation buffer (HBSS without HEPES [Hyclone; Rockford, IL USA], 1 mg/ml Type I Collagenase [Worthington Biochem; Lakewood, NJ USA]) for 3 min at 37^o^ C. Cells were plated in growth media (DMEM/F12 [Gibco; Carlsbad, CA USA], 2.5 mM Glutamine [Hyclone; Rockford, IL USA], 15% horse serum, 50 U/ml Pen/Strep [Cellgro; Manassas, VA USA], 0.25 microgram/ml Fungizone [Invitrogen; Carlsbad, CA USA] and 2 ng/ml bFgf [Upstate/Millipore; Billerica, MA USA] with 20,000 cells/well in 24-well tissue culture plate [Costar; Corning, NY USA] containing type 1 collagen [Sigma; St. Louis, MO USA] coated coverslips for immunocytochemistry or in 35 mm glass bottom culture dish [MatTek; Ashland, MA USA] coated with type 1 collagen for cell tracking assay. Cells were allowed to attach for 1 hr before switching to serum free media containing phenol red free DMEM [Cellgro; Manassas, VA USA], 50 U/ml Pen/Strep [Cellgro; Manassas, VA USA], 0.25 microg/ml Fungizone [Invitrogen; Carlsbad, CA USA], 2.5 mM L-glutamine, 25 mM HEPES, 1 mM Na-pyruvate [Cellgro; Manassas, VA USA].

### Live Cell Tracking Assays

Glass bottom culture dishes containing attached myogenic cells from MMP (Lbx1^EGFP^|Pitx2^+/+^, Lbx1^EGFP^|Pitx2^LacZ/+^, Lbx1^EGFP^|Pitx2^LacZ/LacZ^) and MP (Pax3^Cre^|Rosa^EGFP^|Pitx2^+/+^, Pax3^Cre^|Rosa^EGFP^ |Pitx2^LacZ/+^, Pax3^Cre^|Rosa^EGFP^ |Pitx2^LacZ/LacZ^) cells were taken immediately after switching to serum free media for imaging that persisted for approximately 40 hrs. Culture dishes were placed inside live cell chamber incubator set at 37°C and 5% CO_2_. Single cells were imaged under Zeiss Confocal Microscope LSM 510 Meta [Zeiss; Oberkochen Germany] using EGFP signal as a tracer. The position of individual cells was recorded every 5 min for a total of 2 hrs.

### Immunohistochemistry/Immunocytochemistry

Cryosectioning of fixed E12.5 embryo blocks were cut at 12 microm thickness or myogenic cell culture coverslips were harvested and fixed in 4% Paraformaldehyde containing 0.1% Triton X-100 for 5 min. Samples were washed 3 times with PBS and blocked for 1 hr at room temperature with 3SB blocking buffer (5% Fetal Calf Serum, 5% Goat Serum, 1% Calf Serum, 0.3% Boehringer Blocker, 0.1% Triton X-100, PBS). Primary antibodies anti-mouse Talin (1∶1000, [Sigma; St. Louis, MO USA]), anti-mouse Tropomyosin (undiluted, [DSHB; Iowa City, IA USA]), anti-mouse beta-gal (1∶1000, [Cappel; Cochranville PA USA]), anti-mouse Tau (1∶100, [Santa Cruz; Santa Cruz, CA USA]), anti-mouse alpha-tubulin (1∶1000, [Sigma; St. Louis, MO USA]), anti-rabbit Stmn2 (1∶100, [Abcam; San Francisco, CA USA]), anti-rat BrdU (1∶50 [Accurate Chemical and Scientific West Bury, NY USA] and anti-rabbit desmin (1∶20, [Sigma; St. Louis, MO USA]) added to samples, and samples were incubated overnight at 4°C. Samples were washed 3 times with PBST (PBS +0.1% Triton X-100) for 10 min. Fluorescent conjugated secondary antibodies [1∶500, Jackson Immuno.; West Grove, PA USA] and Alexa Fluor 488 conjugated or Rhodamine conjugated Phalloidin [1∶100, Invitrogen; Carlsbad, CA USA] were added and samples were incubated at room temperature for 2 hrs, followed by 3 times wash with PBST for 10 min. Samples were dehydrated and mounted with DPX mounting media. Single cells were imaged under Zeiss Confocal Microscope LSM 510 Meta [Zeiss; Oberkochen Germany] at 63× magnification. While tissue sections were imaged under Zeiss Imager.Z1 Microscope [Zeiss; Oberkochen Germany] at 63× magnification.

### Visualization of Predicted Gene Network

Cytoscape 2.6.3 was utilized for composing visualizations of microarray gene expression data [65]. The top ten genes were clustered based on fold change, from Table 1 in the families of actin, microtubule, and adhesion. Connections between the nodes are displayed as solid lines for direct interactions and dashed lines for possible interactions. Direct or indirect, not yet determined, was based on current literature search.

## Supporting Information

Figure S1 **Decrease in Number of EGFP^+^ cells in Pitx2 Mutant Forelimbs.** Flow cytometry of dissociated forelimb tissue isolated from E12.5 Pax3^cre/+^|ROSA^EGFP^|Pitx2^LacZ/+^ (HET, n = 8) and Pax3^cre/+^|ROSA^EGFP^|Pitx2^LacZ/LacZ^ (MUT, n = 7) embryos (**A**) Mean (± SEM) number of cells (EGFP^+^ and EGPF^−^ cells combined) from HET tissue was 5,237,143±482,445 cells and MUT tissue was 6,994,000±731,302 cells. (**B**) Mean number of EGFP^+^ cells collected from HET tissue was 877,808±67,469 cells and MUT tissue was 729,630±70,855 cells at a purity of >90%. (**C**) Mean percent of EGFP^+^ cells present in HET forelimb tissue was 17±0.6% and 11±1% in MUT forelimb tissue. This reduced mean percent EGFP+ cells was determined to be significant using unpaired t-test, p = 0.0001.
(TIF)

Figure S2 **Motility Defects in Pax3^+^ Myogenic Cells in Pitx2 Mutants.** Live cell tracking assay of muscle progenitors (n = 5) isolated from E12.5 forelimb tissue of Pax3^cre/+^|ROSA^EGFP^|Pitx2^+/+^ (WT), Pax3^cre/+^|ROSA^EGFP^|Pitx2^LacZ/+^ (HET), or Pax3^cre/+^|ROSA^EGFP^|Pitx2^LacZ/LacZ^ (MUT) embryos. Migration pathways recorded for WT (**A**), HET (**B**) and MUT (**C**). (**D**) Mean total distance travelled of WT (63±14 micrometer), HET (65±14 micrometer) and MUT (34±12 micrometer). (**E**) Mean velocity of movement of WT (1.0±0.6 micrometer/min), HET (0.6±0.2 micrometer/min) and MUT (0.4±0.1 micrometer). (**F**) Mean time spent moving vs. paused for WT moving (76±26 min) and paused (49±26 min), HET moving (76±26 min) and paused (49±26 min) and MUT moving (47±8 min) and paused (78±8 min). Using Dunnett's ANOVA test setting WT as control, MUT MMPs were found to be significantly different in distance travelled, velocity, and time moving vs. paused. Following Dunnett's ANOVA an unpaired T-test between WT and MT determined significance values for distance traveled (p = 0.008), velocity (p = 0.0479), time moving (p = 0.044) and time paused (p = 0.044). (**G**) Quantitation of persistent migratory directionality. Relative ratios of D/T showed that HET cells had a ratio of 98% and MUT cells had a ratio of 167%. (**H**) The mean square displacement of total pathway distance traveled (T^2^) measured every 20 min. The x-intercept for WT (diamonds, black dotted line) and HET (light grey squares, solid light grey line) cells were as close to the origin than the x-intercept for MUT (dark grey triangles, solid dark grey line) cells, indicating that cells from all genotypes exhibit similar migration behaviors.
(TIF)
