# Supplementary figures and images for: Regulation of Motility of Myogenic Cells in Filling Limb Muscle Anlagen by Pitx2
Source: PLoS One. 2012 Apr 27;7(4):e35822. doi: 10.1371/journal.pone.0035822 (PMC3338778; doi:10.1371/journal.pone.0035822)

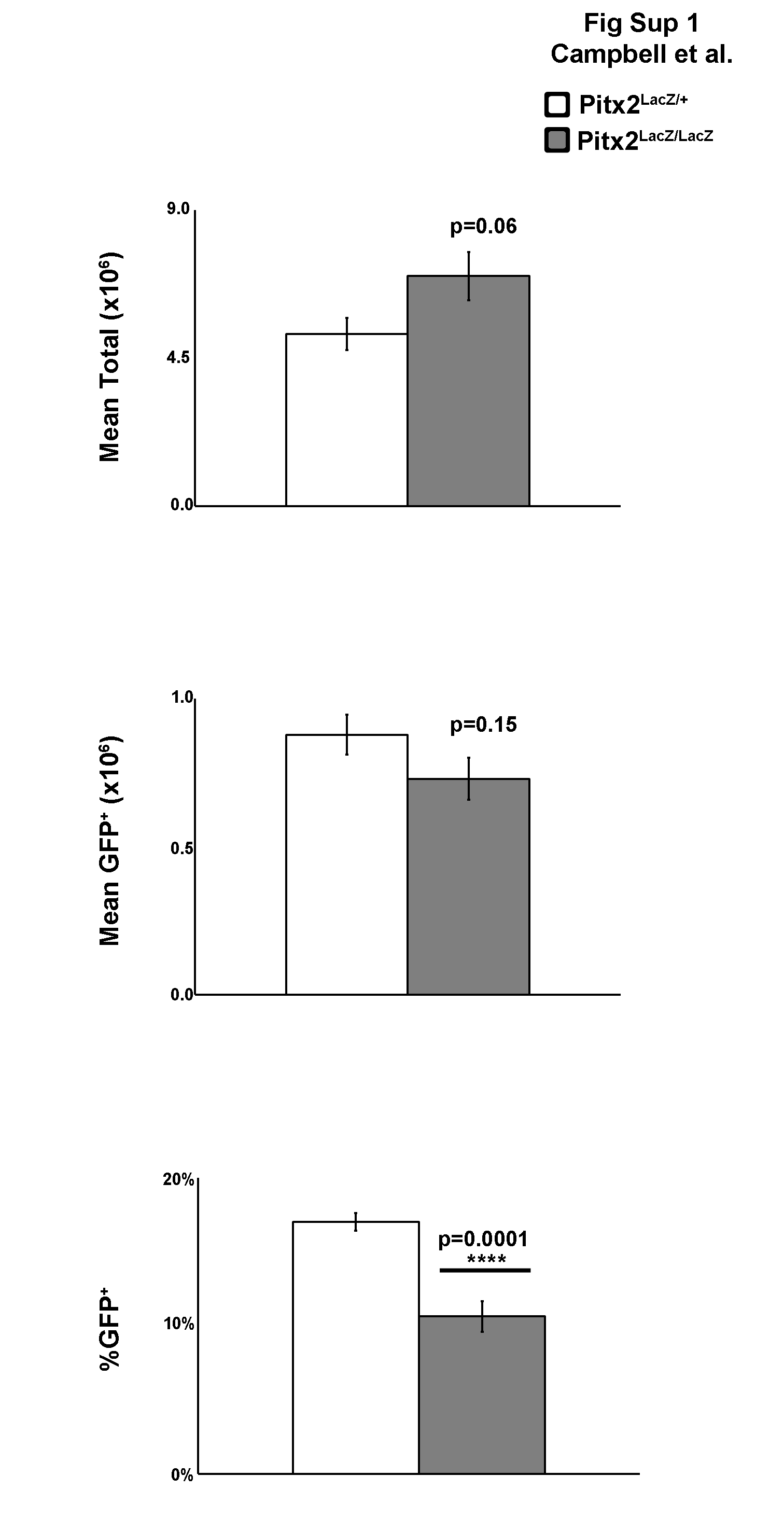

Supplement: Figure S1 — Decrease in Number of EGFP+ cells in Pitx2 Mutant Forelimbs. Flow cytometry of dissociated forelimb tissue isolated from E12.5 Pax3cre/+|ROSAEGFP|Pitx2LacZ/+ (HET, n = 8) and Pax3cre/+|ROSAEGFP|Pitx2LacZ/LacZ (MUT, n = 7) embryos (A) Mean (± SEM) number of cells (EGFP+ and EGPF− cells combined) from HET tissue was 5,237,143±482,445 cells and MUT tissue was 6,994,000±731,302 cells. (B) Mean number of EGFP+ cells collected from HET tissue was 877,808±67,469 cells and MUT tissue was 729,630±70,855 cells at a purity of >90%. (C) Mean percent of EGFP+ cells present in HET forelimb tissue was 17±0.6% and 11±1% in MUT forelimb tissue. This reduced mean percent EGFP+ cells was determined to be significant using unpaired t-test, p = 0.0001. (TIF) [file pone.0035822.s001.tif]

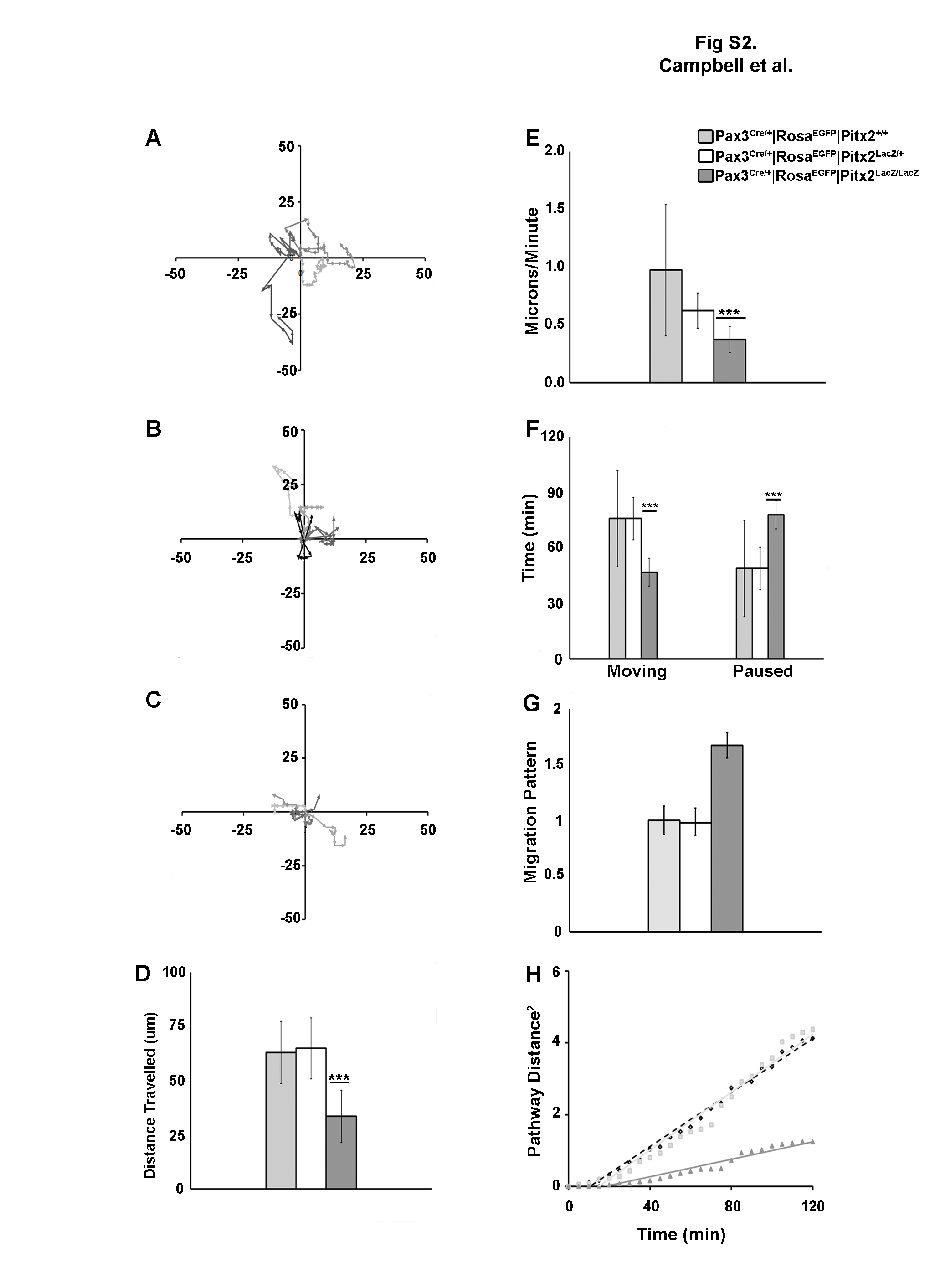

Supplement: Figure S2 — Motility Defects in Pax3+ Myogenic Cells in Pitx2 Mutants. Live cell tracking assay of muscle progenitors (n = 5) isolated from E12.5 forelimb tissue of Pax3cre/+|ROSAEGFP|Pitx2+/+ (WT), Pax3cre/+|ROSAEGFP|Pitx2LacZ/+ (HET), or Pax3cre/+|ROSAEGFP|Pitx2LacZ/LacZ (MUT) embryos. Migration pathways recorded for WT (A), HET (B) and MUT (C). (D) Mean total distance travelled of WT (63±14 micrometer), HET (65±14 micrometer) and MUT (34±12 micrometer). (E) Mean velocity of movement of WT (1.0±0.6 micrometer/min), HET (0.6±0.2 micrometer/min) and MUT (0.4±0.1 micrometer). (F) Mean time spent moving vs. paused for WT moving (76±26 min) and paused (49±26 min), HET moving (76±26 min) and paused (49±26 min) and MUT moving (47±8 min) and paused (78±8 min). Using Dunnett's ANOVA test setting WT as control, MUT MMPs were found to be significantly different in distance travelled, velocity, and time moving vs. paused. Following Dunnett's ANOVA an unpaired T-test between WT and MT determined significance values for distance traveled (p = 0.008), velocity (p = 0.0479), time moving (p = 0.044) and time paused (p = 0.044). (G) Quantitation of persistent migratory directionality. Relative ratios of D/T showed that HET cells had a ratio of 98% and MUT cells had a ratio of 167%. (H) The mean square displacement of total pathway distance traveled (T2) measured every 20 min. The x-intercept for WT (diamonds, black dotted line) and HET (light grey squares, solid light grey line) cells were as close to the origin than the x-intercept for MUT (dark grey triangles, solid dark grey line) cells, indicating that cells from all genotypes exhibit similar migration behaviors. (TIF) [file pone.0035822.s002.tif]
